# Medicinal and useful plants in the tradition of Rotonda, Pollino National Park, Southern Italy

**DOI:** 10.1186/1746-4269-9-19

**Published:** 2013-03-23

**Authors:** Paola Di Sanzo, Laura De Martino, Emilia Mancini, Vincenzo De Feo

**Affiliations:** 1 Dipartimento di Farmacia, Università degli Studi di Salerno, Via Ponte don Melillo, Fisciano, (Salerno) 84084, Italy

**Keywords:** Ethnopharmacology, Traditional medicine, Basilicata region, Calabria region

## Abstract

**Background:**

This paper reports an ethnobotanical survey of the traditional uses of medicinal and useful plants in an area of the Pollino National Park, Basilicata, Southern Italy. The study, conducted between 2009 and 2010, gathered information on the medicinal plants traditionally used in the neighbourhood of town of Rotonda, in the Pollino National Park, that appears have very rich and interesting ethnopharmacological traditions.

**Methods:**

In all, we interviewed 120 key informants, whose age ranged between 50 and 95 years.

**Results:**

The research resulted to the identification of 78 medicinal plants belonging to 46 families. Among the species reported, 59 are used in human medicine, 18 for domestic use, 8 in veterinary medicine. Several plants have been reported in previous studies, but with different uses, or never reported.

**Conclusions:**

Data obtained showed that in the studied area the folk use of plants is alive and still derives from daily practice.

## Background

The plant kingdom represents a source of drugs and foods. Therefore, with the tendency in modern medicine to assimilate and re-assimilate natural remedies in common practice, under various forms, the potential of regional flora becomes important [1]. Systematic exploration of traditional Pharmacopoeias are urgently required in Southern Europe, especially in those areas which, for geographical and historical reasons, remain relatively isolated, and where industrial development has not lead to a complete decline of local traditions [2].

Ethnobotanical studies, supporting the ethno-anthropological sciences, and studies of "material culture", are infrequent in Italy. Generic information is sometimes present in botanical texts concerning handicraft uses, but the plant matter of single artefacts is rarely defined, changing from place to place and originating peculiar local manufacturies. Ethnobotanical uses of plants are often lost more easily in modern civilisation that substitutes traditional handicrafts. This study has been carried out in an area of Basilicata, where, in evident contrast with that happens in the rest of Italy, we can still witness a large folk use of plants and a rich and intense memory of their uses is present. Due to history, economy and tradition, this area could potentially be a precious source of information already lost in other places [3].

Obviously, ethnobotanical studies are very important in those areas, in which the ancient traditions are alive yet. It’s noteworthy underline the value of tradition as something that has been an integrated part of a culture for more than one generation [4]. Within the sphere of research aimed at producing a census of the heritage of usage and folk traditions of useful plants, which today disappear due to an increasing technological lifestyle, ethnobotanical inquiries have been conducted in different parts of Southern Italy, with the purpose of gathering information on traditional medicinal use of plants. In fact, Basilicata Region results partially investigated and some reports on ethnobotanical research are available for this region [5-13]: in particular, a previous study of flora, vegetation and spontaneous food plants underlined the still low industrial and urban impact upon the area of study, considered a good source of information for ethnobotanical applications typical in the central Mediterranean area [14].

In this work, we report about local ethnobotanical uses (for medicinal, veterinary and domestic purposes) of plants in an area of the Pollino National Park in Potenza Province, Basilicata region: this research covers the towns of Rotonda, Villa Meliscio, San Basile, Pedali, and Viggianello. Our aim was to collect the popular knowledge on medicinal plants and their traditional uses to preserve this kind of information that is losing and to evaluate if the collected data can be useful as basis for further phytochemical studies. The available literature shows that such studies can constitute the starting point for the development of new drugs and useful substances [15].

## Methods

### Study area

**Figure 1:**
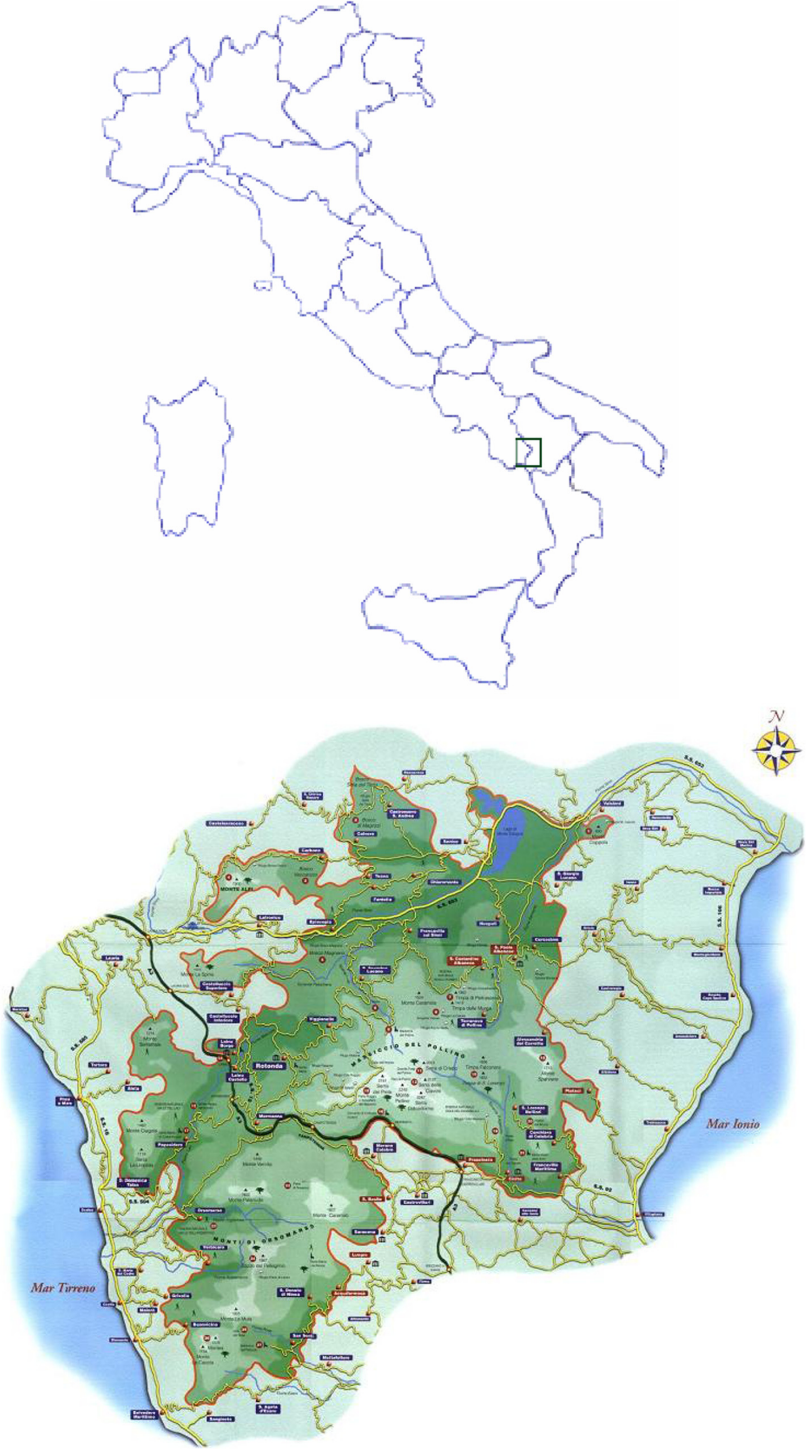
The location of the area studied.

The Pollino National Park (Figure 1) is the biggest natural park in Italy, spreading over more than 2000 km^2^ in areas of absolute wilderness and cultural landscapes in Southern Italy. The Park first aim is preserving the traditional combination of cultural and natural landscapes as well as the unique plant diversity, characteristic for the region. Since the Pollino area has been isolated from the main industrial development until the middle of the 20^th^ century, the traditional plant knowledge is still alive [16,17]. The particular isolation of the mountainous area, which has been the object of our survey, and its economy, which is still partially based on small-scale agricultural and pastoral activities, represents a good opportunity for conducting studies about local of traditional pharmaceutical knowledge. The vegetation is mainly composed by Mediterranean species: at low elevations, the vegetation is mainly constituted by woods of holmoak (*Quercus ilex* L.), interspersed with Mediterranean shrubland (*Quercion ilicis*) and coppices of *Castanea sativa* Miller. A maquis is present in areas more influenced by man, with typical shrubs (*Myrtus communis* L., *Spartium junceum* L., *Viburnum tinus* L.) and small trees (*Arbutus unedo* L., *Quercus pubescens* Willd., *Acer monsplessulanum* L.). Beech (*Fagus sylvatica* L.) woodlands occur at higher elevations, together with *Ilex aquifolium* L. and *Daphne laureola* L. (*Aquifolio Fagetum)* or *Sorbus aucuparia* L., *Sorbus aria* Crantz, *Quercus cerris* L., *Acer pseudoplatanus* L. and *Acer lobelli* Ten. (*Acer lobelii – Fagetum*). In the full area of the Pollino National Park, the presence of 1500 botanical taxa has been hypothesized [16].

### Ethnobotanical methodology

Field data were collected, in several time intervals, during the period 2009–2010 (December 2009-December 2010) and ethnobotanical information on the uses of plants were gathered through semi-structured and structured interviews with persons, who still retain traditional ground knowledge about medicinal and useful plants. In the first phase of the field study, people were asked to freely recall all medicinal plants and other natural remedies that they use or have used in the past. More specific information was recorded later by using structured interviews in which a specifically developed questionnaire was completed [18] (Additional file 1).

People were asked to describe the method of use and preparation of the traditional medical remedies for each folk taxon quoted. During the interviews, several fresh plant specimens or dried samples stocked in a small transportable field herbarium were shown to the interviewed. If a plant was quoted without having any reference in the herbarium, the informant was followed in the field and invited to show the mentioned species [13].

The interviewed informants were 120 (50 men, 70 women), whose ages ranged from 50 to 95 years, and belonged to families which had strong links with traditional activities of the area. Most of the interviewees (90) were aged over 60, of whom 30 were between 60 and 69, 32 between 70 and 79, 26 between 80 and 90, and 2 were over 90 years old. According to an original anthropological observation, women are depositaries of the ‘curative secrets’ of plants, a fact that becomes even more significant when we consider that lands dedicated to gardens and the showing of cereals are inherited through the female line, while only flocks descend through the male line [10]. Among the informants, 15 were farmers, while the remainder mainly building workers, restaurateurs, shepherds and housewives. They all had been living in the area under study for many years. The informants are aware that the information they have provided will be published and that data will be used only for scientific purposes.

The method followed in this study is intermediate between the classic ethnobotanical systematic enumeration and a pharmacological investigation of plant use. This approach uses the merely qualitative data of classical-ethnobotanical-systematic investigation on plants, and the numerical quantitative data of consensus, in keeping with the guides for studies of pharmaceutical ethnology [18-20]. Voucher herbarium specimens were pressed, labelled, dried and deposited in the Herbarium of the Medical Botany Chair at the University of Salerno. Plant classification and nomenclature follows Pignatti [21] and was checked by an update nomenclature (http://www.theplantlist.org).

### Ethnobotanical data analysis

The use value [22], a quantitative method that demonstrates the relative importance of species known locally, was also calculated according to the following formula: UV = U/N, where UV refers to the use value of a species; U to the number of citations per species; and N to the number of informants. Knowing the use value of a plant species may be useful in determining the use reliability and pharmacological features of the related plant [23].

## Results and discussions

The list of the plants claimed as medicinal and their uses are presented in (Additional file 2: Table S1). For each plant, the following information is provided: botanical name and family, voucher specimen number, local name, parts used and a prescription, use value. The research led to the identification of 78 plants, belonging to 46 families, of which the more widely represented are Labiatae (9), Compositae (5), Rosaceae (4) and Leguminosae (4). The great prevalence of plants belong to Angiosperms (63 species to Dicotyledons and 8 species to Monocotyledons); the use of 2 Gymnosperms and 5 Pteridophytes is also reported.

The crude drugs are generally employed in the dried state by using traditional methods of preparation, namely decoction, infusion, tinctures, cataplasms and direct external applications. Some people store the herbal remedies in a house-pharmacy, and often treat not only himself and own family, but also other people that recognize his herbal knowledge.

The comparison of the folk data collected in this study with the ethnomedical literature from contiguous zones of the Pollino National Park [5-13] and with Italian ethomedical revision [24] shows several species with unreported or new or different uses. The plant uses can be divided into three main categories, plants for: medicinal use (59 species), veterinary use (8 species) and domestic use (18 Species).

### Human medicine

**Figure 2:**
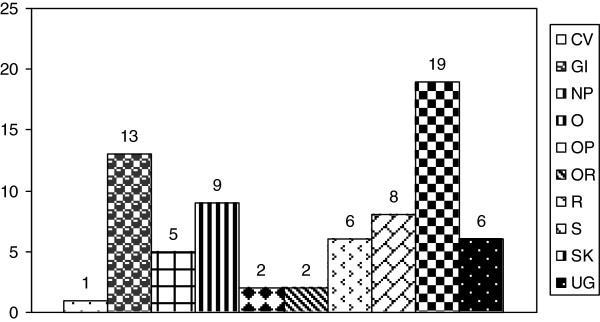
Plants used in human medicine for: cardiovascular diseases (CV); diseases of the gastrointestinal tract (GI); neuropsychiatric diseases (NP); other (O); ophthalmologic diseases (OP); diseases of the oral cavity (OR); diseases of the respiratory system (R); skin diseases (SK); systematic diseases (S); diseases of the urogenital system (UG).

Fifty-nine species, belonging to 38 families, are reported for the human uses. The most cited families are Lamiaceae (7 species), Rosaceae (4 species) and Compositae (4 species). The human uses, according to the traders’ information, are subdivided in ten groups (Figure 2): cardiovascular diseases (CV); diseases of the gastrointestinal tract (GI); neuropsychiatric diseases (NP); ophthalmologic diseases (OP); diseases of the oral cavity (OR); diseases of the respiratory system (R); skin diseases (SK); systematic diseases (S); diseases of the urogenital system (UG); other (O). Among these plants, the highest number is recorded for SK (19 species) and GI (13 species) groups. Less frequently, plant species are used as OR (2 species), OP (2 species) and for CV (1 species). Several species present a new use compared to the literature: a Pteridophythe *Dryopteris filix-mas* (L.) Schott and *Phyllitis scolopendrium* (L.) Newman subsp. *scolopendrium* are used as a lenitive in case of burns. Moreover, a decoction of whole plant of *Adiantum capillus-veneris* L. is claimed to act as a regulator of the menstrual cycle: this use is also reported in literature [9,25]. The decoction of inflorescences of *Sambucus nigra* L. is used as an ocular decongestant. In literature, a similar ophtalmological use of the plant was reported by Leporatti and Impieri [9]. Instead, Pieroni and coworkers [26] reported the use as antifever of the fruit decoction of the plant, confirmed also in other Mediterranean countries [27,28]. Also the uses of *Calystegia sepium* (L.) R. Br. and *Convolvulus arvensis* L. are new in ethnobotanical literature: in fact, both plants are used topically in antirheumatic massages. For the first plant, we found a different use of *C. sepium* that Guarrera and Leporatti [8] reported to promote better and healthier growth in rabbits. For the second plant, the use of this species as digestive was reported [29]. Also the resin of *Abies alba* Miller is used for antirheumatic massages [27]. Pieroni and coworkers [26] reported an antiseptic use of the plant with a topical application. The resin of *A. alba*, prepared in different ways, could be used for skin diseases [30]. Particular is also the use of *Pteridium aquilinum* (L.) Kűhn, of which fronds are employed in antirheumatic mattress. The aerial parts of the plant were used as litter for domestic animals and to make brooms to clean ovens from ashes and sticks [3]. A veterinary use of the plant was reported by Viegi and coworkers [31]: in particular, a decoction of leaves and roots of *Pteridium aquilinum* was used for the expulsion of the placenta in cows. Other uses (antibacterial and diuretic properties) of the leaf decoction were reported [30]. An antihypertensive activity was attributed to the fronds of the plant [32]. We cite the use of a decoction of *Ceterach officinarum* DC. with expectorant property, as already reported in literature [1,29]. In the same region, the species is named "spaccapietre", which means "stone-breaker", and it is typically used in southern Italy for its diuretic properties to treat kidney stones (mineral salts, mucilages and tannins are present) [9]. Instead a decoction of aerial parts was reported to eliminate renal calculus [13]. In Eastern Mallorca (Balearic Islands), a tisane of aerial parts was uses as antihypertensive and hepatic anti-inflammatory [32].

It has to be underlined the use of bulbs fried of *Leopoldia comosa* (L.) Parl.: they are eaten for their diuretic effect. Pieroni and coworkers [13] reported the bulbs of the plant fried with *Capsicum longum* DC. fruits with an antifever effect. The bulbs of the plant were consumed in various ways, flavoured with garlic, chilli pepper, mint and oregano [7]. The grated bulb of *Capsicum* was applied on a paper disc for compresses in case of toothache or headache [8].

The young twigs of *Clematis vitalba* L. are eaten cooked as a diuretic: also the bulbs of the plant were cooked in omelettes [7]. The leaves of the plant were cited by Menković and coworkers [27] for rheumatism. The plant was also used as food in Campania region [25].

In Latium, a soup "acquacotta" was prepared from young shoots of boiled plants such as *Clematis vitalba, Centaurea solstitialis, Scolymus hispanicus, Nasturtium officinale* etc. [8]. Gathering young leaves and buds of wild plants for food purposes is a very ancient custom: the Latin writer Columella describes in "De Agricoltura" that at the spring equinox, various plants were collected: *Clematis vitalba, Ruscus aculeatus, Asparagus* sp. and *Tamus communis* buds and then preserved in vinegar [8]. Salerno and coworkers [3] reported the use of dried stems as a tobacco substitute; the decoction of the fruits was reported as a gargle [10].

In our paper, a decoction of aerial parts of *Centaurium erythraea* Rafn. and of flowering tops of *Teucrium chamaedrys* L. are prescribed internally as a febrifuge, as also a decoction of whole plant of *Cynodon dactylon* (L.) Pers. *C. erythraea* was also reported for its use in skin [29], digestive [27,29,30] and other different disorders [30]. Moreover, the plant is known as an hypotensive and antimalarial. It contains erytro-centaurin, gentiopicrin, fitosterin etc., but it is not known precisely which active principle is responsible for the hypotensive action [8].

*Cynodon dactylon* was also reported for kidney and digestive disorders [29,31,33]. The decoction of roots of the plant was reported as antihaemorrhoidal [30], while the decoction of rhizome was diuretic [10,33]. A syrup of the roots of the plant was known as an antidiarrheal [32]. Guarrera and coworkers reported an alimentary use of *C. dactylon*: infact, the toasted rhizomes of the plant was used to prepare the coffee [7].

*T. chamaedrys* was reported for its medicinal use in Campania region [25] and in other Mediterraenean country [30,32,34]. The decoction of inflorescences of *Tussilago farfara* L. is used as a sedative; instead a decoction of roots, mixed with dried figs, is used as an expectorant. Finally, the leaves of the plant are smoked as an antiasthamtic. The use in the respiratory diseases is also cited in literature [9,27-29]. In González-Tejero and coworkers [29], the plant was cited for its use in muscular-skeletical disorders (in Albania). In treatment of respiratory apparatus diseases, an infusion of flowers and leaves of *Tilia cordata* Miller is used as an antitussive. A tisane of bracts and flowers was reported as tranquilizer [32]. González-Tejero and coworkers [29] cited the use of the plant for respiratory and mental-nervous diseases. The species was also known for the cure of perspiration, diarrohea, stomach spasms and insomnia [34].

Between two plants used for oral cavity, a decoction of whole plant of *Geum urbanum* L. is employed for gargles in case of halitosis.

*Sempervivum tectorum* L. is used in topical applications to counteract headache: in literature [26,30,34] leaf juice of the species, instilled in the ear, was reported to heal pains of the ears. The folk name of the species is "rume", because it is held to help rumination [8,9]. The plant, used to reactivate rumination and as digestive for cattle [9,31] in central Italy, is another example of a potentially interesting species for future veterinary phytotherapy. B2 type procyanidins could be major components of the polymeric polyphenol fraction of fresh leaves of *S. tectorum*[31]. A different use of the plant was reported in different regions of Mediterranean area: the species is reported for treatment of sensory and skin diseases [29].

A decoction of the female cones of *Juniperus communis* L. is claimed to be useful in the treatment of hypertension. An external application of distilled oil of the galbules of the plant was reported as anti-rheumatic [26,30,34]. An use in dyspeptic complaints was reported by others [27,34], as the use for its effects on cardiovascular and muscular-skeletical systems [29].

A particular culinary use of the plant was reported by Jarić and co-workers [34]: berries are used for making and flavouring the brandy known as ‘klekovača’, also used for disinfection owing to antibacterial properties.

The presence of tannins has probably suggested the very particular therapeutic application of *Quercus cerris* L.: in the treatment of haemorrhoids, the patient is seated on the trunk of this plant. The use of the plant is confirmed by Guarrera and coworkers [7], while a different use was reported in Latium region: a cicatrizing decoction was prepared with two handfuls of *Quercus cerris* bark (tannin) and shoots of *Smilax aspera* (1:1) in 10 L of water, boiled down to 2 L [31]. Particular is the use of the wood for barrels in vine production and as fuel for hearths [3].

Fruits of *Cornus mas* L. are used to make astringent jams, as also reported in literature [27,34]. In Croatia, the cherries of the plant were used to produce vinegar [26], as confirmed later [29]. Different preparations of the fruits of the species are employed in Albania for several uses [30]. A domestical use of the species is reported by Salerno and co-workers [3]: in particular, the branches are used for walking sticks for shepherds. With an opposite effect, a decoction of aerial parts of *Borago officinalis* L. is claimed to act as a purgative. The same plant is reported in traditional medicine of other Mediterranean countries with different effect: in particular, in Algeria the plant was used because of muscular-skeletical effect, and in Cyprus island the species was used for respiratory disease [29]. Borage is widely used in Sicily as a diuretic vegetable, which has as well emollient properties on the intestine. In Sardinia the most cited category of use for Borage is that of respiratory ailments like cough and bronchitis [33].

Leaves of *Dipsacus fullonum* L. are used topically in massages, to promote hair growth and strengthening. The latex from fruits of *Solanum melongena* L. is used as an escharotic. The epicarp of the plant, administered in internal way, was used as laxative [32]. A decoction of the flowering tops of *Agrimonia eupatoria* L. is claimed to improve the brain functionality.

For few plants, it is possible to find in the literature some reports that could explain and/or confirm the reported traditional use: for example, the leaves of *Sideritis syriaca* L. are used topically as a vulnerary: in literature, anti-inflammatory and analgesic effects of this plant are reported [9,35] and Leporatti and Impieri [9] cited the use of the hairy leaves of this plant to stop bleeding caused by cuts. A decoction of *Lupinus albus* L*.* is used as a wash to treat dermatitis in cattle in Marche, Toscana and Abruzzo [9]. Leporatti and Impieri [9] recorded an use similar to our also for leaves of *Juglans regia* L.: they reported to put leaves into the shoes against excessive feet perspiration, while we report that a decoction of leaves is used for baths in the same case. Salerno and coworkers [3] reported the use of the decoction of the husk for dyeing dresses black and as hair dye, over the use of wood for veterinary [31] (Viegi et al., 2003) and domestic purposes [25]. The leaves are used as an anti-parasitic when applied to wheat in the granary. This use is widely reported for Marche, Tuscany, northern Latium, Molise, Abruzzo [8] and confirmed by dozens of informants. *J. regia* was cited by more than 30% of informants also in Mustafa and coworkers [30]. Wrapping a cheese with *Juglans regia* leaves repels *Tyrophagus casei*[8]. Other Authors reported several uses of the plant in different regions of Mediterranean area [29,32]. The uses as expectorant and laxative were known. Moreover, an infusion was taken internally as a digestive tonic and for constipation (leaves) and for diarrhoea and anaemia (rind). Applied externally it was used for cuts, grazes and skin disorders such as eczema, herpes, and eruptive skin complain [28,34]. Finally, the husk, macerated in alchol, were used to prepare liqueur nocino [7].

### Veterinary medicine

The use of 8 species, belonging to 7 families, is reported for folk veterinary medicine. Among these species, noteworthy are the uses of 2 Leguminosae: an infusion of fruits of *Lupinus albus* is used as a skin refresher for pigs, whereas a decoction prepared with seeds of bean (*Phaseolus vulgaris* L*.*) is claimed to be a galactophorous for cows.

The poisonous *Veratrum album* L. is used topically in case of mange. Mustafa and coworkers [30] reported different uses of the decoction of leaves and roots: in particular, the first was used as anti-lice, the second one was used for headache. Viegi and coworkers [31] reported an use of the plant in the veterinary medicine.

Besides, for the first time we report the use of *Sambucus ebulus*, administered to cows as a purgative. An interesting medicinal use of the plant is reported for snake bites, by application of the juice from ground leaf [34].

For other 2 species, a new use is reported: *Prunus spinosa* L. is used to prick swollen parts, while *Urtica dioica* L. is used as a galactophorous, (the whole plant) in the veterinary medicine: in other areas the aerial parts of the species are used as a galactophorous in the humane medicine. Pieroni and coworkers [26] reported the leaves of the plant as nutraceuticals, while Guarrera and coworkers [7] recorded the use as fodder. Several veterinary were reported by Viegi and coworkers [31]. Different Authors cited the leaves and the roots of the plant for several different uses as cleansing tonic and blood purifier, fever, arthritis, anaemia, inflammatory diseases of the urinary tract enlarged prostate glands (root). The same plant is used externally, for skin complaints, neuralgia, hemorrhoids, hair problems [10,27,29,30,34]. In Sicily, Leonti and coworkers [33] and Guarrera and Leporatti [8] cited *U. dioica* as a gastrointestinal purifier and anti-diarrhoeic. Leaves are used in form of a cataplasm against haemorrhoids and to prevent hair loss and dandruff [33].

Aerial parts of *Mercurialis annua* L. and *Fraxinus ornus* L. are administered to cows as a purgative; use of *Mercurialis annua* as a purgative for cattles was reported also by Leporatti and Impieri [9]; use of the plant as purgative in Campania, Lucania and in other parts of central and northern Italy [31] should not be encouraged because it contains substances that accumulate in the body (saponine, methylamine, trimethylamine, atractyloside). The use of *Fraxinus ornus* in veterinary and as a laxative is already reported [31].

### Domestic uses

In the Pollino National Park, a few plants are employed also for domestic uses; 18 species, belonging to 13 families. Among domestic uses, 5 plants are used as dyestuff, generally for sheep wool. Among these species, the use of the berries of *Sambucus ebulus* to dye cotton, wool and shoes, appears to be new in the ethnobotanical literature; the fruits are used to prepare ink. Other Authors [3,30] reported some different uses of this species: in particular, they cited the use of flowering top juice to use as writing ink. Also the use of *Solidago virgaurea* L. to dye cloths in yellow appear to be new both for the ethnobotanical literature of Basilicata Region [5,6,8,11-13] and of neighbouring areas [1,36-38]. In Montenegro region, aerial parts of the plant were used to fight inflammation of the urinary tract, kidney stone, nephritis, cystitis, gout [27].

Perhaps, this use can be explained with high flavonoid context of the aerial parts of the plant [39]. *Galium* species, in Rubiaceae family, are used to dye the wool in violet. Although some uses of “domestic” plants appear to be common, others seem to be of interest.

We found 2 species (2 families) used in supporting agricultural practices, for the insect antifeedant activity. In particular, the whole plant of *Ballota nigra* L. is used in repellent fumigation against insects, while the whole plant of *Erodium cicutarium* (L.) L’Hér. is burned as a room insecticide.

The lamiaceous *Ballota nigra* was already reported for its content in diterpenes [40], compounds with well known insecticide and antifeedant activities. *Myrtus communis* L. is employed to tan the hide, probably due to its high content of tannins. In Basilicata and Calabria, the burnt and powdered leaves of *Myrtus* were applied to reddened skin in children and also used to prevent reddening of the feet [10]. The use was widespread in other southern Italian and Mediterranean areas, e.g. Campania, Sicily, Sardinia [8]. The local practice of keeping dried figs skewered and held together with flavouring sticks was also reported for the nearby Mt. Pollino. The aromatizing properties of *Myrtus communis*, containing mirtenol, tannins, terpenes, bitter compound have been known since ancient times: mortadella derives, in fact, from "mortarum", a Roman sausage with myrtle. The salami, ancient "lucànica", seasoned with spices and laurel fruits, of which Apicio (I sec B.C.) describes a recipe, has existed since Roman times [7]. The plant was also employed for its digestive and respiratory effects, in Mediterranean area [29,33]. Its properties for skin diseases were also reported [28,29]. Myrtle was reported in veterinary medicine [31].

Totally new in the ethnobotanical literature is the use of the leaves of *Aristolochia* sp. as a soap substitute in clothes cleaning.

For other plants we cite an uncommon use: the petals of *Papaver rhoeas* are employed in the preparation of a lipstick; *P. rhoeas* was also cited as animal fodder [7] and as ingredient of a mixture vegetable dish, named “misca”, typical of the area and appealing in its simplicity, fit to be served in restaurants and local farm guesthouses [7]. In Mediterranean area, the plant was reported for some different medicinal [29] and veterinary uses [31].

The calyx flower of *Ballota pseudodictamnus* (L.) Bentham was used in past times, and is still used in some isolated areas, as a wick for oil-lamp. Guarrera and Leporatti [8] reported the same use in Apulia and Latium. First microscopic observations revealed that this calyx is constituted by pure cellulose (personal communication, Prof. A.M. Carafa). Moreover, the young branches of *Salix purpurea* L. are used to make hampers, as also reported by Salerno and coworkers [3]. The branches of the plant [27] were used for hemorrhoids. Mustafa and co-workers [30] reported the use of the leaves, that applied topically in breast, had an anti-fever action. Young shoots of *Vitis vinifera* L. are used to clean chimneys. Guarrera and Leporatti [8] reported that young shoots with sap were used as fuel, giving a particular fragrance to the artichokes, cooked according to a local country recipe. Branches of the plant were cited as a tobacco substitute, while the leaves were used to protect for cottage cheese from insects/ dust [3]. Carrió and Vallès [32] reported an external use of a medicinal vinegar of the species for burns. Different uses of the plant were reported in other regions of Mediterranean area [25,29]; Viegi and coworkers [31] reported a veterinary use of the species.

## Conclusions

The study has permitted us to document the traditional knowledge on medicinal plants of the area and to witness, still today, a certain wealth of ethnobotanical information, especially that obtained by interviewing the elderly. The relative isolation of the territory has permitted traditions to be fairly well preserved, which elsewhere have been lost. The uses of medicinal plants connected with ritual prescriptions testifies the good conservation of ancient traditions in this area. In most cases, their applications can be considered rational in the light of modern chemical and pharmacological data.

A great heritage in the field of folk ‘domestic medicine’ may still exist, but most of the remedies quoted in this survey have been abandoned, or are rarely in use today. Only a few of them are still used in the primary health care of the family, normally dispensed by the oldest women of the family. Phytotherapy in this small southern Italian region is today practiced by elderly people who resort now only to medicinal plants for mild illnesses; we interviewed one of them and verified the data with others that, although not recognized as “real” healers, are very experienced in the field.

The present study records new or very scarcely reported medicinal plants and uses, reinforcing the importance of continuing with ethnobotanical research in industrialised areas as a starting point for any bioprospection project, which can lead to the development of new drugs.

Ethnographic, ethnobiological, and ethnopharmacological surveys, dealing with traditional Mediterranean uses of plants and several aspects of folk medicines, could represent the start for the increase of that kind ‘rediscovered’ data concerning on eco-sustainable interdisciplinary projects involving biological conservation, and, most importantly, the conservation of local culture heritage.

## Competing interests

The authors declare that they have no competing interests.

## Authors’ contributions

VDF designed the research project, provided comments and suggestions on the draft. Moreover he conducted the field work, analysed the data and wrote the draft of manuscript. PDS conducted the field work and provided comments on the draft. LDM and EM conducted field work and analysed the data; they have conducted statistical analysis and analysed the manuscript. All authors have read and approved the final manuscript.

## Supplementary Material

Additional file 1 A Questionnaire form for ethnobotanical research. Click here for file

Additional file 2: Table S1 Species traditionally used in the district of Rotonda, in the Pollino National Park. Click here for file
