# Supplementary material for: Medicinal and useful plants in the tradition of Rotonda, Pollino National Park, Southern Italy
Source: J Ethnobiol Ethnomed. 2013 Mar 23;9:19. doi: 10.1186/1746-4269-9-19 (PMC3621777; doi:10.1186/1746-4269-9-19)
Supplement: Additional file 1 — A Questionnaire form for ethnobotanical research. [file 1746-4269-9-19-S1.docx]

APPENDIX A

Questionnaire form for ethnobotanical research

| Vernacular name of the plant |
| --- |
| Used part |
| Harvest time |
| Collection made by:  □ women;  □ men;  □ both  Collecting modality: |
| Employment of the plant:  □ itself;  □ with other plants; in this case, which plants: |
| Preliminary preparation of the plant |
| Vernacular name of culinary preparations |
| Descriptions of above cited preparations |
| Valuation of frequency use of the plant today:  1 □ more times in a week;  2 □ once in a week;  3 □ once in a month;  4 □ twice in a year;  5 □ once in a year or less  Valuation of frequency use of the plant, a time (until 60’s):  1 □ more times in a week;  2 □ once in a week;  3 □ once in a month;  4 □ twice in a year;  5 □ once in a year or less  Valuation of plant appreciation (from two to ten):  □ 8-10: very good;  □ 6-7: fair;  □ 5-6: just edible;  □ 3(or 2)-6: practically unusable  Other uses of the plant (food, medicinal, veterinary, handmade): |
| Potential magic and/or religious uses: |
| Legends and beliefs about plant |
