# Supplementary material for: Medicinal and useful plants in the tradition of Rotonda, Pollino National Park, Southern Italy
Source: J Ethnobiol Ethnomed. 2013 Mar 23;9:19. doi: 10.1186/1746-4269-9-19 (PMC3621777; doi:10.1186/1746-4269-9-19)
Supplement: Additional file 2: Table S1 — Species traditionally used in the district of Rotonda, in the Pollino National Park. [file 1746-4269-9-19-S2.doc]

**TABLE 1**. SPECIES TRADITIONALLY USED IN THE DISTRICT OF ROTONDA, IN THE POLLINO NATIONAL PARK.

| **Family***Species*  (Herbarium number) | **Local names** | **Plant Part used** | **Uses** | **Use value** |
| --- | --- | --- | --- | --- |
| **Adiantaceae** |  |  |  |  |
| *Adiantum capillus-veneris* L. (DF/R/42) | Capiddu venero | Whole plant | **Med.**: A decoction is used internally to regulate the menstrual cycle. | 0.15 |
|  |  |  |  |  |
| **Adoxaceae** |  |  |  |  |
| *Sambucus ebulus* L. (DF/R/75) | Ievolo | Leaves | **Med.**: A decoction is used topically as an antiseptic and an anti-inflammatory in case of wounds. | 0.36 |
|  |  | Dried inflorescences | **Med.**: A decoction is used in case of bronchitis and as a nasal decongestant. |  |
|  |  | Leaves and seeds | **Vet.**: They are administered to cows as a purgative. |  |
|  |  | Berries | **Dom.**: The fruits are used to prepare ink and to dye cotton, wool and shoes. |  |
| *Sambucus nigra* L.  (DF/R/97 | Sammuco | Inflorescences | **Med.**: A decoction is claimed to be a febrifuge in case of bronchitis; topically this preparation is used as an ocular decongestant. | 0.66 |
|  |  | Berries | **Med.**: The fruits are eaten as a laxative. |  |
|  |  |  |  |  |
| **Amaryllidaceae** |  |  |  |  |
| *Allium sativum* L. (DF/R/8) | Agghio | Bulbs | **Dom.**: A maceration is used as a room disinfectant. | 0.46 |
|  |  |  |  |  |
| **Aristolochiaceae** |  |  |  |  |
| *Aristolochia* sp. (DF/R/132) | Edera | Leaves | **Dom.**: The drug is used in water to clean clothes. | 0.03 |
|  |  |  |  |  |
| **Asparagaceae** |  |  |  |  |
| *Leopoldia comosa* (L.) Parl. (DF/R/59) | Cipoddrina | Bulbs | **Med.**: They are eaten fried as a diuretic. | 0.09 |
|  |  |  |  |  |
| *Ruscus aculeatus* L. (DF/R/11) | Rusculu | Young twigs | **Med.**: They are eaten as a diuretic. | 0.11 |
|  |  | Plant | **Dom.**: It is used to clean the chimneys. |  |
|  |  |  |  |  |
| **Aspleniaceae** |  |  |  |  |
| *Ceterach officinarum* DC. (DF/R/163) | Filicetta | Whole plant | **Med.**: A decoction is claimed to be an expectorant. | 0.45 |
|  |  |  |  |  |
| *Phyllitis scolopendrium* (L.) Newman subsp. *scolopendrium* (DF/R/37) | Lengua ‘i cane | Fronds | **Med.**: Used topically as a lenitive in case of burns. | 0.12 |
|  |  |  |  |  |
| **Boraginaceae** |  |  |  |  |
| *Borago officinalis* L. (DF/R/9) | Vorraina | Aerial parts | **Med.**: A decoction is claimed to act as a purgative. | 0.46 |
|  |  |  |  |  |
| **Brassicaceae** |  |  |  |  |
| *Erysimum* sp  (DF/R/28) | Erva ‘i San Francisco | Leaves | **Med.**: The chopped parts are applied topically to resolve abscesses. | 0.11 |
|  |  |  |  |  |
| **Caprifoliaceae** |  |  |  |  |
| *Dipsacus fullonum* L. (DF/R/29) | Spugne | Leaves | **Med.**: The active part is used topically to promote hair growth and strengthening. | 0.27 |
|  |  |  |  |  |
| **Caryophyllaceae** |  |  |  |  |
| *Saponaria officinalis* L.  (DF/R/103) | Saponaria | Aerial parts | **Dom.**: Used in water to clean clothes. | 0.23 |
|  |  |  |  |  |
| **Compositae** |  |  |  |  |
| *Helianthus tuberosus* L.  (DF/R/2) | Tritufolo | Tubers | **Med.**: They are eaten in case of diabetes. | 0.28 |
|  |  |  |  |  |
| *Santolina chamaecyparissus* L.  (DF/R/63) | Musco | Whole plant | **Med.**: The vapour of the plant is used as an expectorant. | 0.12 |
|  |  |  |  |  |
| *Solidago virgaurea* L.  (DF/R/58) | Zimmarino | Inflorescences | **Dom.**: This part is used to dye clothes in yellow. | 0.33 |
|  |  |  |  |  |
| *Taraxacum officinale* Webb  (DF/R/117) | Schirriola | Leaves | **Med.**: A decoction is used as a diuretic. | 0.38 |
|  |  |  |  |  |
| *Tussilago farfara* L.  (DF/R/91) | Calonnedra | Roots | **Med.**: A decoction, mixed with dried figs, is claimed to be an expectorant. | 0.33 |
|  |  | Inflorescences | **Med.**: A decoction is used as a sedative. |  |
|  |  | Leaves | **Med.**: They are smoked as an anti-asthmatic. |  |
|  |  |  |  |  |
| **Convolvulaceae** |  |  |  |  |
| *Calystegia sepium* (L.) R. Br. (DF/R/19) | Velucchio | Aerial parts | **Med.**: The chopped plant is used topically as an antirheumatic. | 0.25 |
|  |  |  |  |  |
| *Convolvulus arvensis* L.  (DF/R/114) | Velucchio | Aerial parts | **Med.**: The chopped plant is used topically as an antirheumatic. | 0.23 |
|  |  |  |  |  |
| **Cornaceae** |  |  |  |  |
| *Cornus mas* L. (DF/R/80) | Cornale | Fruits | **Med.**: These parts are used to make astringent jams. | 0.09 |
|  |  |  |  |  |
| **Crassulaceae** |  |  |  |  |
| *Sempervivum tectorum* L. (DF/R/34) | Siempreviva | Whole plant | **Med.**: The chopped plant is applied to the forehead in case of headache. The same preparation is used topically as an escharotic. | 0.11 |
|  |  |  |  |  |
| *Umbilicus rupestris* (Salisb.) Dandy  (DF/R/101) | Panicucco | Leaves | **Med.**: The chopped parts are used topically as a maturative for abscesses, as a vulnerary and a lenitive in treatment of burns. | 0.14 |
|  |  |  |  |  |
| *Juniperus communis* L. (DF/R/43) | Inipero | Female cones | **Med.**: A decoction is used internally as an antihypertensive. | 0.17 |
|  |  |  |  |  |
| **Dennstaedtiaceae** |  |  |  |  |
| *Pteridium aquilinum* (L.) Kuhn (DF/R/89) | Filici | Fronds | **Med.**: They are use to prepare an antirheumatic mattress. | 0.24 |
|  |  |  |  |  |
| **Dioscoreaceae** |  |  |  |  |
| *Tamus communis* L. (DF/R/106) | Vitella | Tuber | **Med.**: This part is applied topically as an antirheumatic. | 0.09 |
|  |  |  |  |  |
| **Dryopteridaceae** |  |  |  |  |
| *Dryopteris filix-mas* (L.) Schott (DF/R/84) | Filici | Fronds | **Med.**: These, cooked in olive oil, are used as a topic lenitive in case of burns. | 0.22 |
|  |  |  |  |  |
| **Euphorbiaceae** |  |  |  |  |
| *Euphorbia dendroides* L. (DF/R/95) | Cammarone | Latex | **Med.**: This is used as an escharotic, in case of warts. | 0.13 |
|  |  |  |  |  |
| *Mercurialis annua* L.  (DF/R/54) | Mercuredda | Aerial parts | **Vet.**: The aerial parts are administered to cows as a veterinary purgative. | 0.25 |
|  |  |  |  |  |
| **Fagaceae** |  |  |  |  |
| *Quercus* *cerris* L. (DF/R/25) | Cierco | Bark | **Med.**: In the treatment of haemorrhoids, the patient is seated on the trunk drug. | 0.46 |
|  |  |  |  |  |
| **Gentianaceae** |  |  |  |  |
| *Centaurium erythraea* Rafn  (DF/R/22) | Chinino | Aerial parts | **Med.**: A decoction is prescribed internally as a febrifuge. | 0.23 |
|  |  |  |  |  |
| *Gentiana lutea* L.  (DF/R/65) | Genziana | Roots | **Med.**: A decoction is claimed to be an appetizer. | 0.33 |
|  |  |  |  |  |
| **Geraniaceae** |  |  |  |  |
| *Erodium cicutarium* (L.) L’Hér. (DF/R/74) | Erva cimicina | Whole plant | **Dom.:** The plant is burned as a room insecticide. | 0.23 |
|  |  |  |  |  |
| **Hypericaceae** |  |  |  |  |
| *Hypericum perforatum* L. (DF/R/16) | Petricognola | Aerial parts | **Med.**: The parts are macerated in oil, then used as a lenitive in case of burns. | 0.44 |
|  |  |  |  |  |
| **Iridaceae** |  |  |  |  |
| *Iris germanica* L. (DF/R/62) | Spateddra | Rhizome | **Dom.**: The drug is used to scent clothes. | 0.25 |
|  |  |  |  |  |
| **Juglandaceae** |  |  |  |  |
| *Juglans regia* L. (DF/R/6) | Noce | Fruits | **Dom.**: The hull is used to dye the wool in brown. | 0.45 |
|  |  | Leaves | **Med.**: A decoction is used for baths in case of excessive feet perspiration. |  |
|  |  |  |  |  |
| **Lamiaceae** |  |  |  |  |
| *Ballota nigra* L. (DF/R/107) | Marrobbio fetente | Whole plant | **Dom.:** The drug is used in repellent fumigation against insects. | 0.24 |
|  |  |  |  |  |
| *Ballota pseudodictamnus* (L.) Benth.  (DF/R/88) | Miccio | Calyx flower | **Dom.**: The drug is used as a wick for oil night-lamps. | 0.45 |
|  |  |  |  |  |
| *Calamintha nepeta* (L.) Savi  (DF/R/61) | Niepo | Aerial parts | **Med. Or Food**: An infusion is claimed to act as a digestive and a food-flavouring agent. | 0.27 |
|  |  |  |  |  |
| *Marrubium vulgare* L.  (DF/R/78) mont 2012 div | Marrobbio | Flowering aerial parts | **Med.**: An infusion is claimed to be an appetizer. | 0.26 |
|  |  |  |  |  |
| *Mentha aquatica* L.  (DF/R/92) | Mentastica | Leaves | **Med.**: These parts are eaten in salads as digestives. | 0.33 |
|  |  |  |  |  |
| *Mentha pulegium* L.  (DF/R/96) | Poleo, puleio | Plant | **Med.**: The dried drug is chopped and applied topically as an expectorant and for antirheumatic massages. | 0.44 |
|  |  |  |  |  |
| *Sideritis syriaca* L.  (DF/R/66) | Erva janca | Leaves | **Med.**: The active parts are used topically as a vulnerary. | 0.22 |
|  |  |  |  |  |
| *Stachys officinalis* (L.) Trevis. (DF/R/13) | Issopo | Aerial parts | **Med.**: A decoction is claimed to act as a skin and scalp antinfective. | 0.16 |
|  |  |  |  |  |
| *Teucrium chamaedrys* L.  (DF/R/52) | Cersuddra, cerseddra | Whole plant | **Med.**: An infusion is used as an appetizer. | 0.28 |
|  |  | Flowering tops | **Med.**: The decoction is used internally as a febrifuge. |  |
|  |  |  |  |  |
| **Lauraceae** |  |  |  |  |
| *Laurus nobilis* L.  (DF/R/38) | Lauro | Leaves | **Med.**: A decoction is used as a gastric antispasmodic and a general sedative. | 0.44 |
|  |  |  |  |  |
| **Leguminosae** |  |  |  |  |
| *Lupinus albus* L.  (DF/R/57) | Luppino | Fruits | **Vet.**: An infusion is used as a vulnerary, a lenitive and a skin refresher, in particular for pigs. | 0.07 |
|  |  |  |  |  |
| *Ononis viscosa* L.  (DF/R/99) | Erva dei viermi | Aerial parts | **Med.**: A decoction is claimed to be an antispasmodic. | 0.03 |
|  |  |  |  |  |
| *Phaseolus vulgaris* L.  (DF/R/4) | Fasolo | Seeds | **Vet.**: In veterinary a decoction is claimed to be a galactophorous. | 0.33 |
|  |  |  |  |  |
| *Spartium junceum* L.  (DF/R/45) | Sparto | Aerial parts | **Med.**: Chopped parts are used topically as an escharotic. | 0.11 |
|  |  |  |  |  |
| **Malvaceae** |  |  |  |  |
| *Malva sylvestris* L. (DF/R/81) | Malva | Roots and leaves | **Med.**: A decoction is used topically as a lenitive. Orally, the same preparation is claimed to be a febrifuge and an antitussive. | 0.35 |
|  |  |  |  |  |
| *Tilia cordata* Mill.  (DF/R/51) | Tilio | Flowers and leaves | **Med.**: An infusion of these parts is claimed to be an antitussive. | 0.11 |
|  |  |  |  |  |
| **Melanthiaceae** |  |  |  |  |
| *Veratrum album* L. (DF/R/40) | Veletro | Leaves | **Vet.**: The drug is used for baths in case of mange. | 0.08 |
|  |  |  |  |  |
| **Myrtaceae** |  |  |  |  |
| *Myrtus communis* L. (DF/R/53) | Mirtiridda | Whole plant | **Dom.**: It is used to tan hides. | 0.59 |
|  |  |  |  |  |
| **Oleaceae** |  |  |  |  |
| *Fraxinus ornus* L. (DF/R/86) | Mereio | Sap | **Med.**: It is drunk as a laxative. | 0.23 |
|  |  | Flowers | **Vet.**: They are administered to cows as a purgative. |  |
|  |  |  |  |  |
| **Papaveraceae** |  |  |  |  |
| *Papaver rhoeas* L. (DF/R/68) | Paparina | Petals | **Dom.**: They are used in the preparation of lipsticks. | 0.23 |
|  |  |  |  |  |
| **Pinaceae** |  |  |  |  |
| *Abies alba* Mill. (DF/R/118) | Pito | Resin | **Med.**: It is applied topically as a vulnerary and an antiseptic for wounds and for antirheumatic massages. | 0.22 |
|  |  |  |  |  |
| **Plantaginaceae** |  |  |  |  |
| *Plantago* spp. (DF/R/31) | Cinghiniervo | Leaves | **Med.**: A decoction is claimed to be a skin lenitive and used in case of ocular inflammations. | 0.23 |
|  |  |  |  |  |
| **Poaceae** |  |  |  |  |
| *Cynodon dactylon* (L.) Pers. (DF/R/3) | Gramigna | Whole plant | **Med.**: A decoction is used internally as a febrifuge and an antitussive. | 0.46 |
|  |  |  |  |  |
| *Zea mays* L.  (DF/R/77) | Trucchisco | Stigmas | **Med.**: A decoction is used as a diuretic. | 0.33 |
|  |  |  |  |  |
| **Portulacaceae** |  |  |  |  |
| *Portulaca oleracea* L.  (DF/R/35) | ‘Ntracchia | Plant | **Med.**: The drug is eaten as depurative in salads. | 0.19 |
|  |  |  |  |  |
| **Ranunculaceae** |  |  |  |  |
| *Clematis vitalba* L.  (DF/R/69) | Grampuddrina | Young twigs | **Med.**: They are eaten cooked as a diuretic. | 0.07 |
|  |  |  |  |  |
| *Ranunculus ficaria* L.  (DF/R/93) | Ricchia gattina | Leaves | **Med.**: They are applied topically as a lenitive. | 0.03 |
|  |  |  |  |  |
| **Rosaceae** |  |  |  |  |
| *Agrimonia eupatoria* L. (DF/R/14) | Agrimonia | Flowering tops | **Med.**: A decoction is used to improve brain functionality. | 0.23 |
|  |  |  |  |  |
| *Geum urbanum* L. (DF/R/67) | Bella ‘i notte | Whole plant | **Med.**: A decoction is used for gargles in case of halitosis. | 0.12 |
|  |  |  |  |  |
| *Prunus spinosa* L.(DF/R/83) | Prugnolo | Thorns | **Vet.**: They are used to prick swollen parts. | 0.09 |
|  |  | Young branches | **Med.**: The young parts are used topically as a lenitive and an antinfective. |  |
|  |  |  |  |  |
| *Rubus* sp. (DF/R/64) | Sipali, spiazzo | Young branches | **Med.**: A decoction is claimed to be an antitussive. | 0.25 |
|  |  |  |  |  |
| **Rubiaceae** |  |  |  |  |
| *Galium odoratum* (L.) Scop.  (DF/R/79) | Miscarola | Plant | **Dom.**: The drug is used to dye the wool in violet. | 0.14 |
|  |  |  |  |  |
| *Galium verum* L.  (DF/R/60) | Miscarola | Plant | **Dom.**: The drug is used to dye the wool in violet. | 0.17 |
|  |  |  |  |  |
| **Rutaceae** |  |  |  |  |
| *Ruta graveolens* L.(DF/R/33) | Ruta | Aerial parts | **Med.**: They are chopped and then applied topically in pork fat as an antirheumatic and in case of sciatica and headache. | 0.29 |
|  |  |  |  |  |
| **Salicaceae** |  |  |  |  |
| *Salix purpurea* L. (DF/R/41) | Salicone | Young branches | **Dom.**: The active parts are used to make hampers. | 0.35 |
|  |  |  |  |  |
| **Scrophulariaceae** |  |  |  |  |
| *Verbascum thapsus* L. (DF/R/21) | Verbasco | Flowers | **Med.**: An infusion is used in treatment of colds. | 0.22 |
|  |  |  |  |  |
| **Solanaceae** |  |  |  |  |
| *Hyoscyamus niger* L. (DF/R/105) |  | Seeds and flowers | **Med.**: They are applied topically in case of toothache. | 0.09 |
|  |  |  |  |  |
| *Solanum melongena* L.  (DF/R/17) | Mirlignana | Fruits | **Med.**: They are used topically as an escharotic. | 0.27 |
|  |  |  |  |  |
| **Urticaceae** |  |  |  |  |
| *Parietaria officinalis* L. (DF/R/12) | Erva viento | Whole plant | **Dom.**: It is used to clean glass utensils. | 0.25 |
|  |  | Whole plant | **Vet.**: A decoction is used as a lenitive. |  |
|  |  |  |  |  |
| *Urtica dioica* L.  (DF/R/5) | Lurdica | Roots | **Med.**: A decoction is used internally as an antirheumatic. | 0.44 |
|  |  | Plant | **Vet.**: It is used, topically, for galactophorous massages. |  |
|  |  |  |  |  |
| **Verbenaceae** |  |  |  |  |
| *Verbena officinalis* L.  (DF/R/48) | Verbena | Aerial parts | **Med.**: A decoction is claimed to be an appetizer. | 0.23 |
|  |  |  |  |  |
| **Vitaceae** |  |  |  |  |
| *Vitis* *vinifera* L.  (DF/R/44) | Vita | Young shoots | **Dom.**: They are used to clean the chimneys. | 0.45 |

**Med.:** Plants used in human medicine; **Vet.:** Plants used in veterinary medicine; **Dom.:** Plants used for domestic use.
